# *ACSL4*-driven ferroptosis susceptibility as a targetable vulnerability in monocytic acute myeloid leukemia

**DOI:** 10.3389/fonc.2026.1837940

**Published:** 2026-05-22

**Authors:** Federico De Marchi, Michele Gottardi

**Affiliations:** Department of Hematology, Veneto Institute of Oncology IOV – IRCCS, Padua, Italy

**Keywords:** *ACSL4*, acute myeloid leukemia, dasatinib, ferroptosis, monocytic differentiation, SRC kinase, venetoclax resistance

## Abstract

Ferroptosis, an iron-dependent form of regulated cell death driven by lethal lipid peroxidation, has emerged as a targetable vulnerability in cancer. *ACSL4* is the rate-limiting enzyme that dictates ferroptosis sensitivity by channeling polyunsaturated fatty acids into membrane phospholipids. In acute myeloid leukemia (AML), monocytic subtypes resist BCL2 inhibition with venetoclax, yet their metabolic dependencies remain poorly defined. Here, we integrated PRISM drug-sensitivity data (6,790 compounds) and DepMap CRISPR dependencies (18,435 genes) across 15 adult AML cell lines to map drug-gene co-dependencies. A multi-cohort validation strategy — filtering 50 discovery candidates (29 testable in BeatAML) through *ex vivo* drug-response data in 476 primary AML specimens and clinical outcomes in 140 adults with *de novo* AML (TCGA-LAML) — converged on a single axis linking SRC family kinase inhibition to *ACSL4* expression. *ACSL4*-high blasts showed enhanced dasatinib sensitivity (*r* = -0.25, *P* = 4.3 x 10^-8^). A composite *SRC*/*ACSL4* signature stratified overall survival (HR 1.27; 95% CI 1.10-1.47; *P* = 0.0014), remaining significant after age adjustment. Single-cell atlas projection localized this signature to the monocytic compartment. The *SRC*/*ACSL4*-high state displayed a ferroptosis gene expression profile characterized by co-upregulation of *ACSL4*, *HMOX1*, and *LPCAT3* with failure to upregulate the principal ferroptosis defense axis *GPX4*/*SLC7A11*. Conversely, *ACSL4*-high blasts showed significant *ex vivo* resistance to venetoclax (*r* = 0.36, *P* = 2.5 x 10^-12^), linking the ferroptosis-primed monocytic state to BCL2 inhibitor failure. These findings nominate *ACSL4*-driven ferroptosis susceptibility as a lineage-specific vulnerability rendering monocytic AML selectively sensitive to SRC-directed therapy while resistant to BCL2 inhibition.

## 1 Introduction

Ferroptosis is a regulated form of non-apoptotic cell death driven by iron-dependent accumulation of lipid peroxides within cellular membranes (1, 2). Unlike apoptosis, which proceeds through caspase-mediated pathways, ferroptosis is governed by the balance between pro-oxidant lipid substrates and the glutathione peroxidase 4 (GPX4) antioxidant system (3). Central to this balance is acyl-CoA synthetase long-chain family member 4 (*ACSL4*), the rate-limiting enzyme that esterifies polyunsaturated fatty acids (PUFAs) — particularly arachidonic and adrenic acid — into membrane phospholipids, thereby creating the oxidizable substrate pool required for ferroptotic death (4). Cells with high *ACSL4* activity maintain a PUFA-enriched phospholipid landscape that is intrinsically poised for iron-catalyzed peroxidation, rendering them ferroptosis-susceptible — a state that has been termed “ferroptosis priming” (5).

The therapeutic relevance of ferroptosis priming has been demonstrated in solid tumors, where drug-tolerant persister cells and therapy-resistant mesenchymal states display paradoxical vulnerability to GPX4 inhibition (5, 6). However, the role of *ACSL4*-driven ferroptosis susceptibility in hematologic malignancies — and specifically in acute myeloid leukemia (AML) — remains largely unexplored.

Treatment of AML in older adults has been transformed by venetoclax-based BCL2 inhibition (7), yet a critical barrier to durable remissions has emerged: AML with monocytic differentiation exhibits intrinsic resistance to venetoclax, reflecting a metabolic shift away from BCL2 dependence toward alternative survival circuits (8). This monocytic reservoir is now recognized as a major mechanism of venetoclax treatment failure and relapse. Monocytic cells are characterized by active lipid remodeling and inflammatory signaling programs, but whether these features create actionable ferroptosis-related vulnerabilities has not been systematically investigated.

We hypothesized that integrative pharmacogenomics could reveal ferroptosis-linked dependencies specific to the monocytic AML compartment. To test this, we developed a stepwise screening strategy integrating drug-sensitivity and CRISPR dependency data from adult AML cell lines, validated candidates in primary patient specimens with functional drug-response readouts, evaluated prognostic significance in an independent clinical cohort, and mapped the resulting signature onto a single-cell hematopoietic atlas and the ferroptosis gene regulatory landscape. This approach identified a convergent axis linking *ACSL4* to SRC family kinase (SFK) dependence, localizing a ferroptosis-primed, pharmacologically targetable state to the monocytic lineage.

## 2 Materials and methods

### 2.1 Data sources

Pharmacologic sensitivity profiles (area under the curve, AUC) for 6,790 compounds were obtained from the PRISM Repurposing 24Q2 dataset (9). Genome-wide CRISPR gene-dependency scores (Chronos scores for 18,435 genes) were retrieved from the Cancer Dependency Map (DepMap) 24Q2 release (10). Both datasets were restricted to the 15 adult AML cell lines profiled in both resources.

For translational validation, *ex vivo* drug-response profiles and matched RNA-seq data from 476 primary AML specimens were obtained from the BeatAML cohort (11). Clinical outcomes and RNA-seq expression data for 140 adults with *de novo* AML were retrieved from the TCGA-LAML cohort (12). Single-cell transcriptional profiles were obtained from a recently published atlas of human bone marrow hematopoiesis (13).

### 2.2 Discovery of drug-gene co-dependencies

We implemented a linear association modeling framework (LAMARCA) to identify drug-gene co-dependencies. For each compound *d* with annotated on-target gene *g_on_*, a base model was fitted: AUC*_d_* = *beta*_0_ + *beta*_1_ x Chronos(*g_on_*) + *epsilon*. Residuals were correlated (Spearman) with CRISPR dependency scores for all 18,435 genes to nominate candidate co-target genes. For each candidate *g_co_*, a combined model incorporating both the on-target and co-target dependency was fitted, and interactions were prioritized by gain in explained variance (Delta-R^2^ = R^2combo^ - R^2base^), controlled for multiple testing (Benjamini-Hochberg FDR < 0.01), and assessed by partial Spearman correlation controlling for the primary on-target effect.

### 2.3 Robustness assessment

All candidate interactions were assessed by three complementary robustness metrics: (i) permutation testing (200 random shuffles of drug-sensitivity labels to establish an empirical null distribution), (ii) leave-one-line-out (LOLO) cross-validation (fraction of 15 leave-one-out iterations in which the interaction remained positive), and (iii) random specificity testing (ensuring each co-target gene was not a nonspecific predictor of unrelated drug sensitivities). Robustness metrics for all interactions are reported in **Supplementary Table 1**.

### 2.4 BeatAML translational validation

Each pharmacologic probe from the discovery set was mapped to its closest clinical analog in BeatAML. For each candidate drug-gene pair, a linear model tested whether expression of the nominated co-target predicted *ex vivo* drug response (AUC) in primary blasts, with and without the on-target gene as a covariate. Partial correlations assessed the independent predictive contribution of the co-target. Results were corrected for multiple testing (FDR < 0.05).

### 2.5 Survival analysis

**Figure 1:**
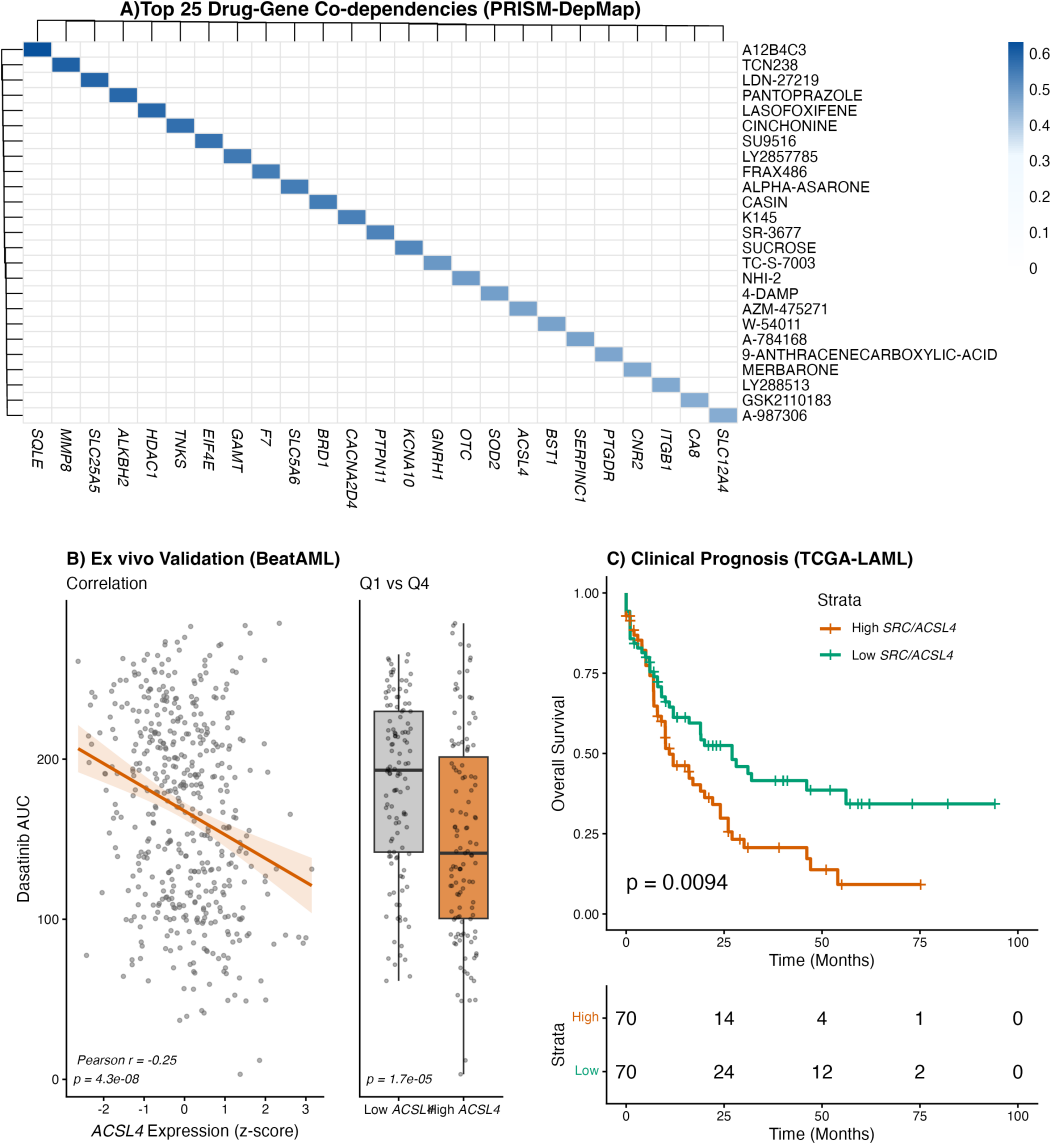
Identification and validation of the SRC-ACSL4 ferroptosis axis in adult AML. **(A)** Heatmap of the top 25 high-confidence drug-gene co-dependencies identified by integrating PRISM drug-sensitivity and DepMap CRISPR dependency data across 15 adult AML cell lines. Rows represent individual drugs ranked by Delta-R^2^; columns represent the nominated co-target gene for each interaction. Color intensity reflects interaction signal strength (Pearson correlation between drug sensitivity residual and co-target CRISPR dependency). All interactions satisfied FDR < 0.01; permutation, leave-one-line-out, and random specificity robustness metrics are reported in **Supplementary Table 1**. ACSL4 appears as co-target for two drugs: AZM-475271 (SRC inhibitor) and indeglitazar (PPAR agonist). **(B)** *Ex vivo* validation in primary AML blasts (BeatAML, n = 476). Left: scatter plot depicting the inverse correlation between ACSL4 expression (z-score) and dasatinib AUC (Pearson *r* = -0.25, *P* = 4.3 x 10^-8^), indicating that ACSL4-high blasts are preferentially sensitive to dasatinib. Right: comparison of dasatinib AUC between the lowest (Q1, ACSL4-low) and highest (Q4, ACSL4-high) expression quartiles (*P* = 1.7 x 10^-5^). **(C)** Clinical evaluation in the TCGA-LAML cohort (n = 140). Kaplan-Meier curves of overall survival stratified by composite *SRC*/*ACSL4* linear predictor (median split) demonstrate significantly inferior survival in the *SRC*/*ACSL4*-high group (log-rank *P* = 0.0094). Cox regression: HR 1.27 (95% CI 1.10-1.47; *P* = 0.0014); age-adjusted HR 1.18 (95% CI 1.01-1.37; *P* = 0.032).

The SRC-ACSL4 gene pair was nominated entirely from independent data (PRISM/DepMap discovery and BeatAML validation); no survival data were used in candidate selection. In TCGA-LAML (n = 140, 87 events), a composite linear predictor (LP) was constructed as the sum of z-scored *SRC* and *ACSL4* expression, with equal weighting for each gene. Patients were stratified into high and low groups by median LP for Kaplan-Meier analysis (log-rank test). The LP was then modeled as a single continuous variable in univariable and age-adjusted Cox proportional-hazards regression. To assess independence from established molecular risk factors, multivariable Cox models additionally adjusted for *FLT3*, *NPM1*, *TP53*, *IDH1*, and *IDH2* mutation status (FLT3 and NPM1 from PCR-based clinical annotations; *TP53*, *IDH1*, and *IDH2* from whole-exome sequencing non-silent mutation calls), and cytogenetic risk category (favorable, intermediate, or poor). Univariable Cox models and time-dependent receiver-operating-characteristic analyses at 1, 2, and 3 years (*timeROC* package) were fitted for *ACSL4*, *SRC*, and the combined LP separately. The independent contribution of *ACSL4* to *ex vivo* dasatinib sensitivity was assessed in the same BeatAML validation set used for **Figure 1B** (n = 476) using a joint linear model and partial Pearson and Spearman correlations of *ACSL4* and *SRC* against dasatinib AUC. The LP, *ACSL4*, and *SRC* z-scores were compared between mutated and wild-type AML for *FLT3*, *NPM1*, *TP53*, *IDH1*, and *IDH2* (BeatAML clinical annotations and WES non-silent mutation calls) using Wilcoxon rank-sum tests with Benjamini-Hochberg FDR control. Because the LP and survival outcomes were evaluated within the same cohort, the survival results should be interpreted as evidence of clinical relevance rather than externally validated prediction.

### 2.6 Single-cell atlas projection

A composite *SRC*/*ACSL4* score was defined as the sum of z-scored *SRC* and *ACSL4* expression and projected onto a single-cell transcriptional atlas of normal human bone marrow hematopoiesis (13). Mean signature scores were computed per annotated cell type to determine which hematopoietic lineage the *SRC*/*ACSL4*-high state maps to.

### 2.7 Ferroptosis gene landscape

To characterize the ferroptosis regulatory state of *SRC*/*ACSL4*-high AML, we examined the correlation (Spearman rho) between the *SRC*/*ACSL4* LP and expression of established ferroptosis effector and defense genes across 22,360 genes in TCGA-LAML. Key genes were selected based on their well-characterized roles in ferroptosis execution (*ACSL4*, *LPCAT3*, *ALOX5*, *HMOX1*), iron metabolism (*FTH1*, *FTL*, *TFRC*, *NCOA4*), canonical antioxidant defense (*GPX4*, *NFE2L2*), alternative ferroptosis defense pathways (*AIFM2*/FSP1, *GCH1*, *DHODH*), and cystine import (*SLC7A11*, *SLC3A2*).

### 2.8 Pathway and transcription factor analysis

Gene set enrichment analysis (GSEA) was performed using Hallmark and REACTOME gene sets from MSigDB (14) on genes ranked by correlation with the *SRC*/*ACSL4* LP. Transcription factor activity was estimated using DoRothEA regulon-based inference (15), and activity scores were correlated with the LP to identify the regulatory network underlying the *ACSL4*-high state.

### 2.9 Statistical analysis

All analyses were performed in R (v4.3). Correlations were assessed using Pearson and Spearman coefficients. Group comparisons used two-sample t-tests or Wilcoxon rank-sum tests as appropriate. Survival analyses used the *survival* and *survminer* packages. Multiple testing correction employed the Benjamini-Hochberg procedure. All P values are two-sided.

## 3 Results

### 3.1 Integrative pharmacogenomic screen identifies *ACSL4* as a recurrent co-dependency

Application of the LAMARCA framework to PRISM drug-sensitivity and DepMap CRISPR dependency data across 15 adult AML cell lines yielded 50 high-confidence drug-gene co-dependencies satisfying stringent thresholds (Delta-R^2^ > 0.02, FDR < 0.01). These were further assessed by permutation testing (all empirical *P* < 0.05), leave-one-line-out cross-validation, and random specificity controls; robustness metrics for all interactions are reported in **Supplementary Table 1** (**Figure 1A**).

*ACSL4* emerged as a co-target for two mechanistically distinct drugs: AZM-475271, an SRC kinase inhibitor (Delta-R^2^ = 0.48, partial rho = -0.82, *P* = 1.9 x 10^-4^), and indeglitazar, a PPAR receptor agonist targeting *NCOA1* (Delta-R^2^ = 0.43, partial rho = -0.75, *P* = 1.3 x 10^-3^). The independent convergence of two pharmacologically unrelated compounds on *ACSL4* as a co-dependency gene supports its biological significance as a metabolic node in AML and implicates the PPAR-*ACSL4* lipid-metabolic axis as a broader vulnerability.

### 3.2 Translational validation converges on the *SRC*-*ACSL4* axis

Of the 50 discovery interactions, 29 had a testable drug analog in the BeatAML cohort (n = 476 primary specimens with matched *ex vivo* drug-response and RNA-seq data). This translational filter reduced the candidate pool to two interactions significantly associated with *ex vivo* drug sensitivity (FDR < 0.05): an axis linking SRC inhibition to *ACSL4* expression (probed with dasatinib; FDR = 2.2 x 10^-5^, partial rho = -0.21, *P* = 2.7 x 10^-6^) and an interaction between *GSK3B* inhibition and *NAGK* expression (probed with LY294002; FDR = 0.017). The *SRC*-*ACSL4* axis was retained as the primary finding based on stronger statistical significance, larger sample size (n = 476 versus n = 81), and direct mechanistic relevance to ferroptosis.

*ACSL4* expression showed a significant inverse correlation with dasatinib AUC (Pearson *r* = -0.25, *P* = 4.3 x 10^-8^), indicating that *ACSL4*-high blasts are preferentially sensitive to dasatinib, a multi-kinase inhibitor with potent SRC family kinase activity (**Figure 1B**). Comparison of *ACSL4* expression quartiles confirmed significantly lower AUC (greater sensitivity) in Q4 versus Q1 (*P* = 1.7 x 10^-5^). A joint linear model of dasatinib AUC on z-scored *ACSL4* and *SRC* expression was fitted in the same BeatAML validation set used for **Figure 1B** (n = 476; **Supplementary Table 3**) to determine whether the *ACSL4*-dasatinib association reflects *SRC* co-expression. *ACSL4* remained associated with dasatinib sensitivity after adjustment for *SRC* (joint-model coefficient -13.5; partial Pearson *r* = -0.22, *P* = 8.7 x 10^-7^), with an effect size approximately 2.3-fold larger than that of *SRC* (joint coefficient -5.8; partial *r* = -0.10, *P* = 0.032). *ACSL4* therefore captures information about *ex vivo* dasatinib response that is not explained by *SRC* abundance and is best interpreted as a marker of a distinct metabolic state. The PRISM discovery identified *ACSL4* as a co-dependency for AZM-475271, annotated as a selective SRC inhibitor; dasatinib was used for BeatAML validation as the only SRC-targeting compound available in that cohort. As a specificity control, *ACSL4* expression was tested against *ex vivo* sensitivity to five non-SRC kinase inhibitors in BeatAML; dasatinib remained the strongest and most significant association, with no signal for ALK, FLT3/Aurora, or ATP-competitive AKT inhibitors, arguing against a generic kinase-sensitivity phenotype (**Supplementary Figure 1**).

### 3.3 The *SRC*/*ACSL4* signature stratifies clinical outcomes

**Figure 2:**
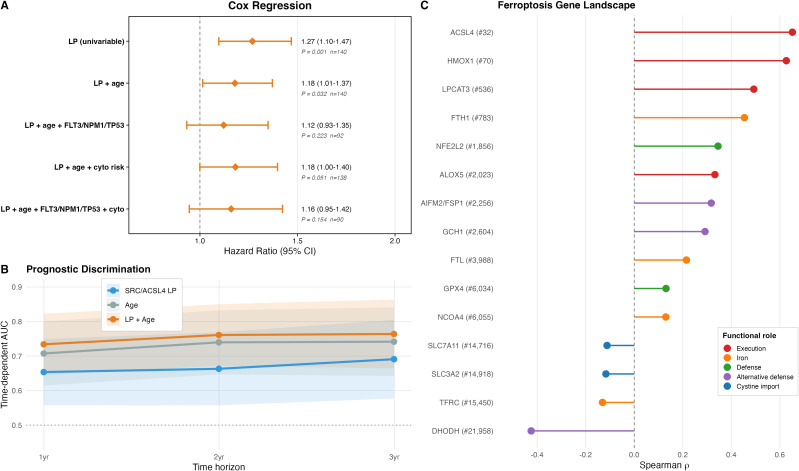
Prognostic model performance and ferroptosis gene landscape. **(A)** Forest plot of Cox proportional-hazards regression for the *SRC*/*ACSL4* LP across five progressively adjusted models. The LP is significant in univariable (HR 1.27, *P* = 0.0014) and age-adjusted (HR 1.18, *P* = 0.032) models, borderline after cytogenetic risk adjustment (HR 1.18, *P* = 0.051, n = 138), and attenuated in models incorporating molecular markers (n = 92 with complete annotation). **(B)** Time-dependent AUC for overall survival prediction at 1, 2, and 3 years. The *SRC*/*ACSL4* LP alone (blue) achieves AUC 0.65-0.69; age alone (gray) achieves AUC 0.71-0.74; the combined LP + age model (orange) achieves AUC 0.73-0.76, indicating that the molecular signature contributes independent prognostic information additive to age. **(C)** Ferroptosis gene expression landscape in TCGA-LAML. Lollipop plot showing Spearman correlation (rho) between the *SRC*/*ACSL4* linear predictor and expression of key ferroptosis-related genes across 22,360 genes. Genes are grouped by functional role: ferroptosis execution (*ACSL4*, *LPCAT3*, *HMOX1*, *ALOX5*), iron metabolism (*FTH1*, *FTL*, *NCOA4*, *TFRC*), canonical defense (*GPX4*, *NFE2L2*), alternative defense (*AIFM2*/FSP1, *GCH1*, *DHODH*), and cystine import (*SLC7A11*, *SLC3A2*). Genome-wide rank shown in parentheses. The *SRC*/*ACSL4*-high state exhibits co-upregulation of ferroptosis-executing genes with failure to upregulate *GPX4* (rank 6,034), downregulation of the xCT cystine transporter (*SLC7A11* rank 14,716), and strong downregulation of *DHODH* (rank 21,958), defining a ferroptosis-primed state with inadequate engagement of all three major defense systems despite partial FSP1/GCH1 compensation.

The clinical relevance of this axis was evaluated in the TCGA-LAML cohort (n = 140, 87 events). The composite linear predictor (LP) is the sum of z-scored *SRC* and *ACSL4* expression, with each gene contributing equal weight: LP = *z*(SRC) + *z*(ACSL4). High LP values therefore identify patients in whom both transcripts are co-elevated; the score is given on a per-patient basis and was not derived from outcome data. The *SRC*/*ACSL4*-high group (median split) experienced significantly inferior overall survival (log-rank *P* = 0.0094; **Figure 1C**), and Cox regression confirmed the adverse prognostic impact of the LP as a continuous variable (HR 1.27; 95% CI 1.10-1.47; *P* = 0.0014), remaining significant after adjustment for age (HR 1.18; 95% CI 1.01-1.37; *P* = 0.032) and borderline after additional adjustment for cytogenetic risk category (HR 1.18; 95% CI 1.00-1.40; *P* = 0.051; n = 138). In models incorporating *FLT3*, *NPM1*, and *TP53* mutation status (n = 92 with complete molecular annotation), the LP was attenuated (HR 1.12; 95% CI 0.93-1.35; *P* = 0.22); in the most fully adjusted model (n = 90), HR was 1.16 (95% CI 0.95-1.42; *P* = 0.15), reflecting both shared prognostic information with established risk markers and reduced statistical power in the smaller annotated subset (**Figure 2A**; **Supplementary Table 2**).

To compare the prognostic contribution of each gene with the combined signature, univariable Cox models and time-dependent AUCs were fitted for *ACSL4*, *SRC*, and the LP separately (**Supplementary Figure 3**). *SRC* expression alone carried the dominant signal (HR 1.58 per z-unit, 95% CI 1.24-2.00, *P* = 1.7 x 10^-4^; log-rank *P* = 8 x 10^-4^). *ACSL4* alone was not significantly associated with overall survival in TCGA-LAML (HR 1.14, 95% CI 0.92-1.40, *P* = 0.24; log-rank *P* = 0.32). The combined LP (HR 1.27, *P* = 0.0014; log-rank *P* = 0.0094) sat between the two single-gene predictors, and the corresponding time-dependent AUCs at 1, 2, and 3 years showed the same ordering, with the LP plus age providing the highest discrimination (**Figure 2B**, **Supplementary Figure 3D**). The LP does not outperform *SRC* expression as a stand-alone prognostic biomarker; its rationale is biological. *ACSL4* defines the ferroptosis-primed metabolic state and predicts dasatinib response in primary specimens (§3.2); *SRC* identifies the targetable kinase dependence. The combined signature carries both halves of the axis and is intended as a marker of the SRC-dependent monocytic ferroptosis-primed state, not as a survival score in its own right.

We next asked whether the *SRC*/*ACSL4*-high state corresponds to known molecular AML subgroups. The LP, *ACSL4*, and *SRC* were tested against *FLT3*, *NPM1*, *TP53*, *IDH1*, and *IDH2* mutation status in both cohorts (**Supplementary Figure 4**; **Supplementary Table 2**). TCGA-LAML mutation-positive subgroups were too small for definitive comparison. In BeatAML (n = 595–647 per gene with paired expression and mutation calls), the LP was significantly lower in *IDH1*- and *IDH2*-mutated cases (LP: *IDH1*-mut median -0.81 vs WT + 0.11, *P* = 6.1 x 10^-4^, FDR = 0.009; *IDH2*-mut median -0.49 vs WT + 0.10, *P* = 1.5 x 10^-3^, FDR = 0.011), and *ACSL4* and *SRC* were each individually lower in both *IDH* groups (FDR < 0.05). *NPM1*-mutated cases had higher *ACSL4* but no LP shift (*P* = 0.12). *FLT3*-ITD and *TP53* status had no measurable effect on the LP in either cohort. The multivariable Cox model in TCGA-LAML was extended to include *IDH1* and *IDH2* alongside age, *FLT3*, *NPM1*, and *TP53* (n = 91 with complete annotation; **Supplementary Table 2**). *IDH1* and *IDH2* both showed protective trends consistent with prior literature, and the LP estimate did not change relative to the model without *IDH* (HR 1.07, 95% CI 0.87-1.30, *P* = 0.53). The signature is therefore not a proxy for any single mutational subgroup, but is depleted in *IDH*-mutated, primitive AML, in line with a monocytic-lineage signature rather than a progenitor-lineage one (§3.5).

### 3.4 The *SRC*/*ACSL4*-high state displays a ferroptosis-primed gene expression landscape

To directly characterize ferroptosis susceptibility, we examined the expression of established ferroptosis effector and defense genes as a function of the *SRC*/*ACSL4* LP across 22,360 genes in TCGA-LAML (**Figure 2C**).

Ferroptosis-executing genes were among the most positively correlated transcripts in the entire transcriptome. Because *ACSL4* is a component of the LP, its high ranking (32nd, rho = 0.65) is expected and partly tautological. The informative finding is that genes *not* included in the LP — and therefore independently correlated — also point to ferroptosis. *LPCAT3*, which catalyzes the downstream remodeling of PUFA-CoA into membrane phospholipids, ranked 536th (rho = 0.49). *HMOX1*, which releases catalytic free iron from heme to fuel the Fenton reaction, was the 70th most correlated gene genome-wide (rho = 0.63). *ALOX5*, a lipoxygenase that directly catalyzes lipid peroxidation, was also positively correlated (rho = 0.33, rank 2,023). The co-upregulation of *ACSL4*, *LPCAT3*, and *HMOX1* indicates maximal generation of oxidizable PUFA-phospholipid substrates with active iron release — three independent arms of the ferroptosis execution pathway.

By contrast, the principal ferroptosis defense mechanisms were not correspondingly upregulated. *GPX4*, the sole enzyme that reduces lipid hydroperoxides within membranes and the central brake on ferroptosis, ranked only 6,034th (rho = 0.13) — effectively unchanged relative to the LP. The cystine/glutamate antiporter system xCT (*SLC7A11*/*SLC3A2*), which imports cystine for glutathione synthesis and represents the upstream metabolic input to GPX4, was negatively correlated with the LP (*SLC7A11* rho = -0.11, rank 14,716; *SLC3A2* rho = -0.12, rank 14,918). This reduction in xCT expression would be expected to constrain glutathione biosynthesis, further weakening the GPX4-dependent antioxidant shield. *NFE2L2*/NRF2, the master transcription factor of the antioxidant response, showed modest positive correlation (rho = 0.35, rank 1,856), consistent with a compensatory but insufficient stress response.

Beyond the canonical GPX4 axis, we examined alternative ferroptosis defense systems. *AIFM2*/FSP1, the CoQ10-dependent ferroptosis suppressor, showed moderate positive correlation (rho = 0.32, rank 2,256), and *GCH1*, the rate-limiting enzyme of the BH4 antioxidant pathway, was similarly positioned (rho = 0.29, rank 2,604). However, *DHODH*, the mitochondrial ferroptosis defense enzyme, was among the most negatively correlated genes genome-wide (rho = -0.42, rank 21,958 of 22,360), indicating active downregulation in the *SRC*/*ACSL4*-high state (**Figure 2C**). Thus, while partial compensatory engagement of FSP1 and GCH1 is evident, the overall ferroptosis defense remains insufficient: the canonical GPX4-xCT axis is not upregulated, and the mitochondrial DHODH pathway is downregulated.

The *SRC*/*ACSL4*-high transcriptome thus exhibits the hallmarks of a ferroptosis-primed state: maximal PUFA-phospholipid substrate generation (*ACSL4*/*LPCAT3*), active iron-dependent and enzymatic lipid peroxidation machinery (*HMOX1*/*ALOX5*), and inadequate engagement of all three major ferroptosis defense systems (GPX4-xCT, FSP1/CoQ10, and DHODH).

### 3.5 Ferroptosis priming localizes to the monocytic compartment

**Figure 3:**
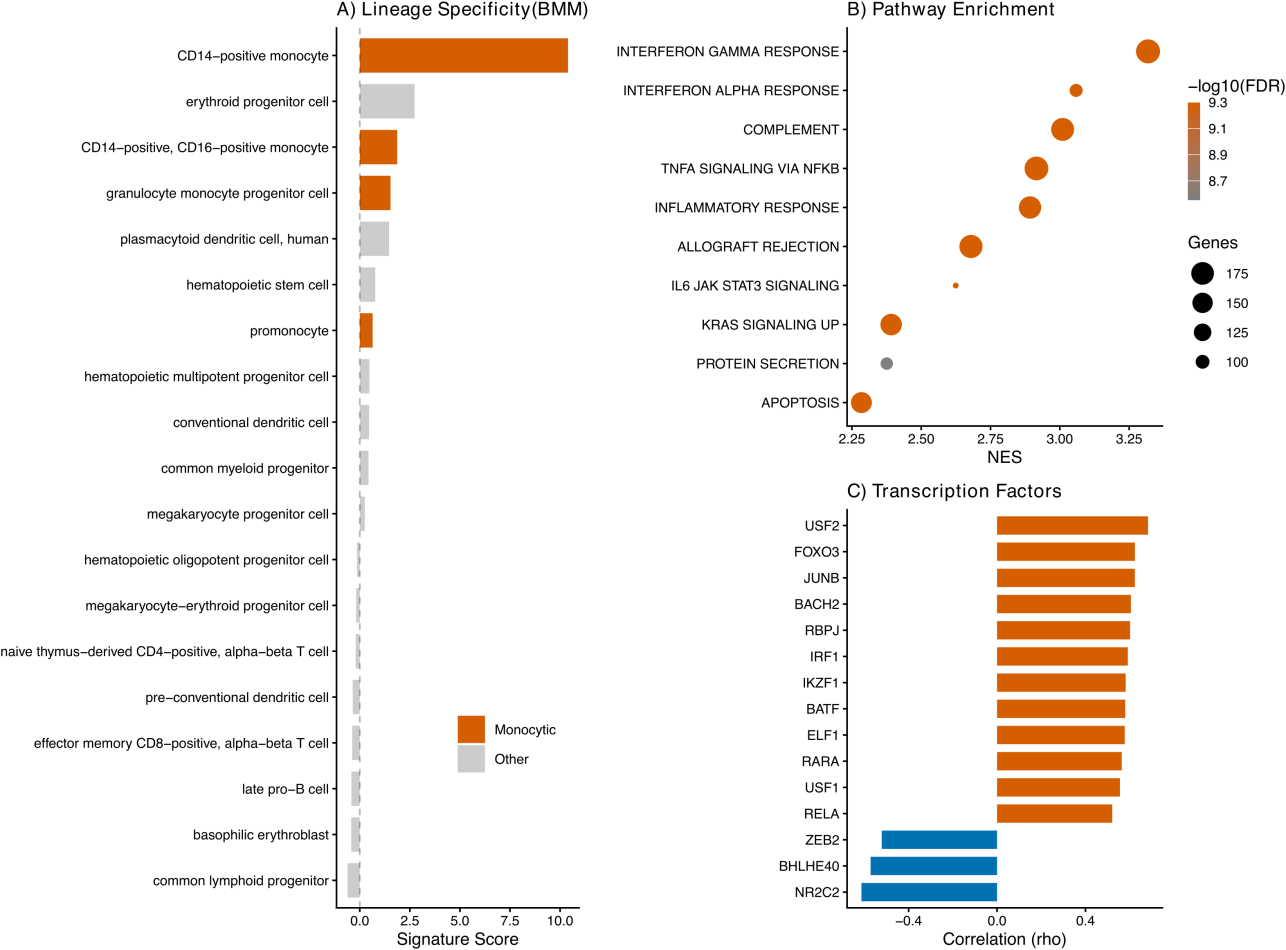
The *SRC*/*ACSL4* ferroptosis signature maps to an inflammatory monocytic regulatory network. **(A)** Projection of the composite *SRC*/*ACSL4* signature score (sum of z-scored expression) onto a single-cell transcriptional atlas of normal human bone marrow hematopoiesis (BMM, Bone Marrow Map; 13). The signature is enriched in monocytic populations (orange): CD14-positive monocytes (score 10.4), erythroid progenitors (2.7), CD14+/CD16+ monocytes (1.9), and granulocyte-monocyte progenitors (1.5). Minimal signal is observed in hematopoietic stem cells (0.77) and lymphoid populations (all negative). **(B)** Gene set enrichment analysis (GSEA) using MSigDB Hallmark gene sets in TCGA-LAML, ranked by normalized enrichment score (NES). The *SRC*/*ACSL4*-high state is dominated by interferon-gamma response (NES = 3.32), interferon-alpha response (NES = 3.06), complement (NES = 3.01), TNF-alpha/NF-kappaB (NES = 2.92), and inflammatory response (NES = 2.89). Dot size = gene set size; color = -log10(FDR). **(C)** DoRothEA transcription factor activity profiling. Top 15 transcription factors by absolute Spearman correlation with the *SRC*/*ACSL4* LP. Positive (orange): activity increases in *SRC*/*ACSL4*-high disease; negative: activity decreases. Key regulators include SPI1/PU.1 (monocytic commitment), IRF1 (interferon/ferroptosis), JUNB (inflammatory AP-1), and NF-kappaB1/RELA (survival).

Projection of the composite *SRC*/*ACSL4* score onto a single-cell transcriptional atlas of normal human bone marrow hematopoiesis (13) revealed clear monocytic specificity (**Figure 3A**). CD14-positive monocytes exhibited the highest signature score (10.4), followed by erythroid progenitors (2.7), CD14+/CD16+ monocytes (1.9), and granulocyte-monocyte progenitors (1.5). Scores were minimal in hematopoietic stem cells (0.77), multipotent progenitors (0.48), and lymphoid populations (all negative). The erythroid progenitor signal is consistent with known *ACSL4* expression in erythropoiesis, where it contributes to membrane lipid remodeling during terminal differentiation; however, the dominant monocytic enrichment (10.4 vs. 2.7, approximately 4-fold) supports lineage-restricted monocytic vulnerability as the primary biology driving this axis.

### 3.6 Inflammatory regulatory network of the ferroptosis-primed monocytic state

Gene set enrichment analysis using Hallmark gene sets confirmed that the *SRC*/*ACSL4*-high state was most strongly associated with interferon-gamma response (NES = 3.32, FDR < 10^-9^), interferon-alpha response (NES = 3.06), complement (NES = 3.01), TNF-alpha signaling via NF-kappaB (NES = 2.92), and inflammatory response (NES = 2.89) (**Figure 3B**). REACTOME analysis independently confirmed enrichment for neutrophil degranulation (NES = 2.98), interferon-gamma signaling (NES = 2.95), Toll-like receptor cascades (NES = 2.78), and Fc-gamma receptor-dependent phagocytosis (NES = 2.70). Negatively enriched pathways included eukaryotic translation and ribosome biogenesis (NES < -3.0), consistent with a shift from proliferative to effector programs.

Transcription factor activity profiling via DoRothEA (**Figure 3C**) identified *USF2* (rho = 0.68), *FOXO3* (rho = 0.62), *JUNB* (rho = 0.62), and *IRF1* (rho = 0.59) as the most positively correlated regulators. *SPI1*/PU.1 — the master transcription factor of monocytic differentiation — was positively correlated (rho = 0.50), as were *RELA* and *NFKB1* (rho = 0.52 and 0.51, respectively). Negatively correlated transcription factors included *NR2C2*, *BHLHE40*, and *ZEB2*, associated with stem-cell maintenance and quiescence. This regulatory architecture positions *ACSL4*-high monocytic AML at the intersection of myeloid differentiation, inflammatory signaling, and ferroptosis susceptibility.

## 4 Discussion

This study identifies *ACSL4*-driven ferroptosis susceptibility as a lineage-specific vulnerability in monocytic AML that is pharmacologically targetable through SRC family kinase inhibition. Through integrative pharmacogenomic and primary-specimen drug-response analysis, we demonstrate that a convergent axis linking *ACSL4* to SRC dependence defines a biologically coherent, clinically adverse, and therapeutically actionable monocytic state.

The ferroptosis gene landscape provides the most direct evidence for this model. *SRC*/*ACSL4*-high blasts co-upregulate the ferroptosis execution machinery (*ACSL4*, *LPCAT3*, *HMOX1*) while failing to proportionally engage any of the three major defense systems: the canonical *GPX4*-xCT axis remains flat, the mitochondrial *DHODH* pathway is downregulated, and partial FSP1/*GCH1* compensation is insufficient. This imbalance — maximal substrate loading with inadequate antioxidant defense — defines a cell dependent on compensatory survival signals to avoid ferroptotic death.

We propose that SRC family kinases provide this compensatory signal in monocytic AML. SRC inhibition would therefore destabilize a precarious equilibrium, selectively triggering ferroptosis in the monocytic compartment while sparing *ACSL4*-low stem and progenitor populations. This model is further supported by the convergence of *ACSL4* as a co-dependency gene for two pharmacologically unrelated compounds — an SRC inhibitor and a PPAR agonist — in the discovery screen, indicating that *ACSL4* is a genuine metabolic node rather than an artifact of a single drug class.

The monocytic specificity of this vulnerability has a physiological basis. Normal monocytes require *ACSL4* for arachidonic acid esterification into membrane phospholipids (4) — the same substrate pool used for eicosanoid synthesis — and constitutively express *HMOX1* for heme catabolism. These are physiological necessities that create an inherent ferroptosis liability, amplified rather than *de novo* acquired during leukemogenesis. In normal bone marrow, CD14-positive monocytes exhibited a signature score of 10.4 versus 0.77 for hematopoietic stem cells (13-fold enrichment), confirming lineage specificity. The Zeng et al. (13) atlas encompasses both normal hematopoiesis and AML differentiation; our projection onto normal cell-type annotations demonstrates that this vulnerability is lineage-inherent, consistent with monocytic AML recapitulating normal monocyte transcriptional programs (8). A direct corollary is that the signature reported here is not restricted to leukemic blasts: the same transcriptional state is expected in normal CD14-positive monocytes from healthy donors, and any therapeutic strategy exploiting *ACSL4*-driven ferroptosis priming will engage normal monocytes and tissue macrophages as well. Defining the therapeutic window will therefore require side-by-side functional comparison of ferroptosis sensitivity between malignant monocytic blasts and their normal counterparts. The mutational analysis is consistent with this lineage interpretation: the LP is depleted in *IDH1*- and *IDH2*-mutated AML, subtypes biased toward primitive rather than monocytic differentiation, and is unrelated to *FLT3*-ITD or *TP53* status, indicating that the signature tracks myeloid maturation rather than any single driver mutation.

The *SRC*/*ACSL4*-high state identifies patients with poor prognosis under intensive chemotherapy (HR 1.27; *P* = 0.0014; TCGA-LAML) and, because this axis maps to the monocytic compartment implicated in venetoclax resistance, we tested whether *ACSL4*-high blasts also resist BCL2 inhibition. In BeatAML, *ACSL4* expression showed a significant positive correlation with venetoclax AUC (*r* = 0.36, *P* = 2.5 x 10^-12^, n = 356; **Supplementary Figure 2**), confirming preferential venetoclax resistance *ex vivo*. Whether this translates to inferior clinical outcomes under venetoclax-based therapy remains to be confirmed in prospectively treated cohorts (16). At the same time, this venetoclax-resistant population is selectively vulnerable to SRC inhibition *ex vivo*, raising the possibility that combining venetoclax (targeting BCL2-dependent primitive AML) with a selective SRC inhibitor (targeting the ferroptosis-primed monocytic reservoir) could provide complementary coverage across the leukemic hierarchy. Biomarker-guided enrichment for the *ACSL4*-high monocytic state may be necessary to realize therapeutic benefit from SRC-directed approaches.

Several limitations warrant explicit discussion. The most important is that the present study is computational throughout. No functional ferroptosis readouts are provided: lipid peroxidation (e.g., C11-BODIPY), labile iron (FerroOrange), GPX4 enzymatic activity, and dose-response to ferroptosis inducers (RSL3, erastin) and rescue agents (ferrostatin-1, liproxstatin-1, deferoxamine) in *ACSL4*-modulated AML models — coupled with *ACSL4* gain- and loss-of-function — are required to demonstrate the inferred phenotype causally. These experiments are the subject of separate follow-up work. The discovery panel comprised 15 AML cell lines, the complete adult AML intersection of PRISM and DepMap at the 24Q2 release; this constraint was mitigated by stringent robustness filters and by independent validation in 476 primary specimens. The LP was attenuated in multivariable models incorporating *FLT3*, *NPM1*, *TP53*, and *IDH1*/*IDH2* mutation status (n = 91-92), reflecting both shared prognostic information with established risk markers and the smaller annotated subset; TCGA clinical annotations record *FLT3* mutation status without distinguishing ITD from TKD (17). Dasatinib is a multi-target kinase inhibitor; although AZM-475271 is a selectively annotated SRC inhibitor in PRISM and the BeatAML negative-control panel showed no *ACSL4* association with ALK, FLT3, or Aurora kinase inhibitors, direct testing with a selective SRC inhibitor in primary AML specimens would further consolidate the on-target interpretation. Finally, the ferroptosis-primed signature is a property of the monocytic lineage and is therefore present to some degree in normal CD14-positive monocytes. Whether *ACSL4*-driven ferroptosis induction can be exploited in monocytic AML without unacceptable on-target toxicity to normal monocytes and tissue macrophages requires direct functional comparison between malignant and normal myeloid populations and lies outside the scope of the present Brief Research Report.

In conclusion, convergent evidence from pharmacogenomic screening, primary specimen validation, and clinical outcome analysis supports a model in which *ACSL4* expression marks a ferroptosis-primed, SRC-dependent monocytic state that is simultaneously vulnerable to SRC inhibition and resistant to venetoclax. SRC-directed therapy, guided by *ACSL4* expression as a biomarker, may open a new therapeutic axis in monocytic AML.

## Data Availability

Publicly available datasets were analyzed in this study. The data presented in the study are deposited in the following repositories: TCGA-LAML (RNA-seq and clinical data): NCI Genomic Data Commons, project accession TCGA-LAML (https://portal.gdc.cancer.gov/projects/TCGA-LAML); dbGaP study accession phs000178 for primary sequencing data. BeatAML (ex vivo drug-sensitivity, RNA-seq and WES mutation data; waves 1–4 release): open-access interactive portal at https://www.vizome.org/aml2/; open-data files at https://github.com/biodev/beataml2.0_data; primary sequencing data in dbGaP under study accession phs001657 (https://www.ncbi.nlm.nih.gov/projects/gap/cgi-bin/study.cgi?study_id=phs001657.v3.p1). DepMap PRISM Repurposing Secondary Screen and DepMap CRISPR dependency (Avana/Achilles), release 24Q2: DepMap Portal (https://depmap.org/portal/data_page/?tab=allData). Single-cell normal-hematopoiesis Bone Marrow Map (Zeng et al., 2025): raw and processed scRNA-seq in GEO, accession GSE289435 (https://www.ncbi.nlm.nih.gov/geo/query/acc.cgi?acc=GSE289435); interactive cellxgene viewer at https://cellxgene.cziscience.com/e/cd2f23c1-aef1-48ae-8eb4-0bcf124e567d.cxg/; reference atlas object and BoneMarrowMap R package at https://github.com/andygxzeng/BoneMarrowMap. All derived data supporting the conclusions are provided in **Supplementary Tables S1**–**S3**. Further inquiries can be directed to the corresponding author.
