# Supplementary material for: ACSL4-driven ferroptosis susceptibility as a targetable vulnerability in monocytic acute myeloid leukemia
Source: Front Oncol. 2026 May 22;16:1837940. doi: 10.3389/fonc.2026.1837940 (PMC13236526; doi:10.3389/fonc.2026.1837940)
Supplement: Supplementary file 1 [file DataSheet1.pdf]

Supplementary Material

ACSL4−Driven Ferroptosis Susceptibility as a Targetable Vulnerability in Monocytic Acute Myeloid Leukemia

Manuscript ID 1837940 − Frontiers in Pharmacology, Pharmacology of Anti−Cancer Drugs

Revision 2 (response to Reviewer 3) − May 2026

Contents

**Supplementary Figure S1**	ACSL4 vs ex vivo sensitivity to six kinase inhibitors in BeatAML (drug−target specificity).
**Supplementary Figure S2**	Venetoclax sensitivity vs the SRC/ACSL4 linear predictor (BCL2 axis specificity control).
**Supplementary Figure S3**	Univariable Kaplan−Meier curves and time−dependent AUC for ACSL4, SRC, and the combined linear predictor (TCGA−LAML).
**Supplementary Figure S4**	Linear−predictor distribution stratified by FLT3, NPM1, TP53, IDH1, and IDH2 mutation status (TCGA−LAML and BeatAML).
**Supplementary Table S1**	LAMARCA−identified drug−gene co−dependencies in adult AML cell lines (top 50 by Delta R^2).
**Supplementary Table S2**	TCGA−LAML Cox proportional hazards models for overall survival, extended Cox coefficients (LP + age + FLT3 + NPM1 + TP53 + IDH1 + IDH2), and Wilcoxon rank−sum tests of LP / ACSL4 / SRC by mutation status (3 pages).
**Supplementary Table S3**	ACSL4 vs SRC independence in BeatAML (n = 476): joint linear model, partial Pearson and Spearman correlations of dasatinib AUC ~ ACSL4 + SRC.

All figures and tables in this bundle were re−derived from the analysis scripts for Revision 2 and re−rendered to address the formatting and content poin

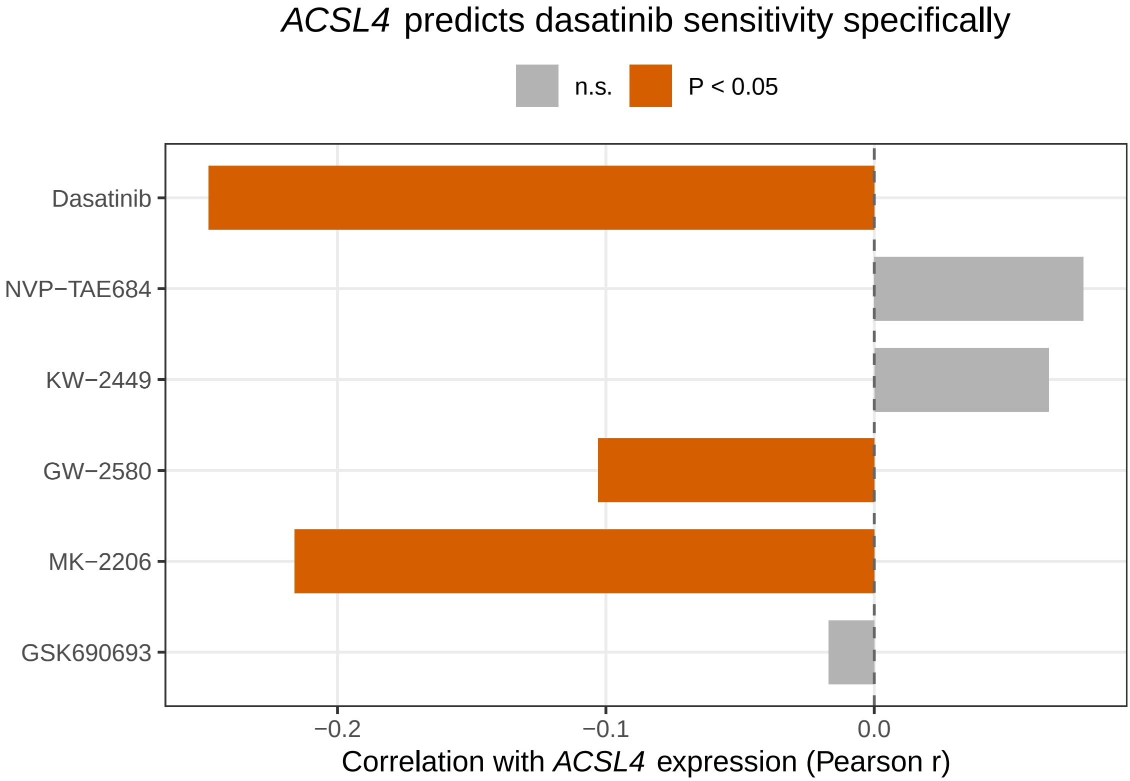
ACSL4 predicts dasatinib sensitivity specifically
n.s.
P < 0.05
Dasatinib
NVP−TAE684
KW−2449
GW−2580
MK−2206
GSK690693
−0.2
−0.1
0.0
Correlation with ACSL4 expression (Pearson r)

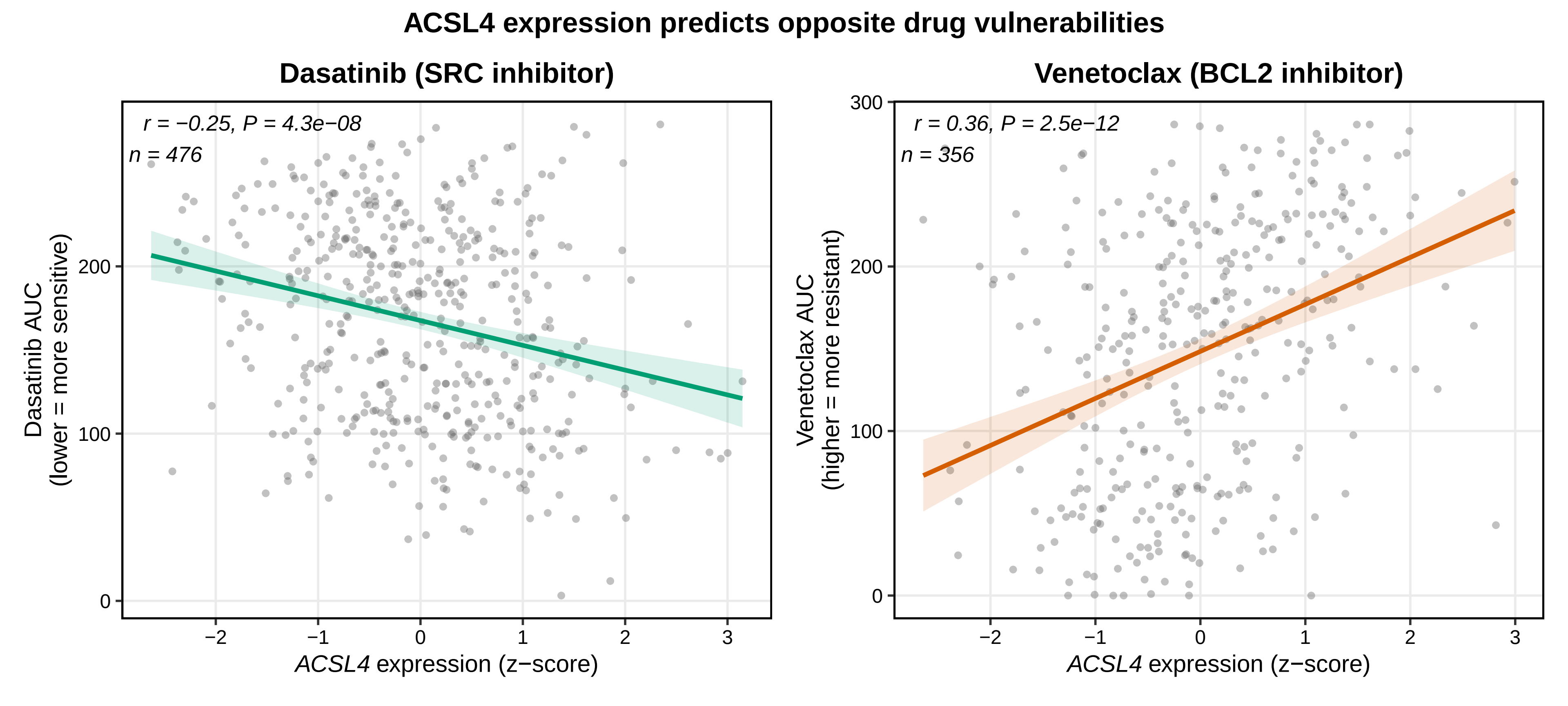

ACSL4 expression predicts opposite drug vulnerabilities
Dasatinib (SRC inhibitor)
r = −0.25, P = 4.3e−08
n = 476
Dasatinib AUC
(lower = more sensitive)
ACSL4 expression (z−score)
Venetoclax (BCL2 inhibitor)
r = 0.36, P = 2.5e−12
n = 356
Venetoclax AUC
(higher = more resistant)
ACSL4 expression (z−score)

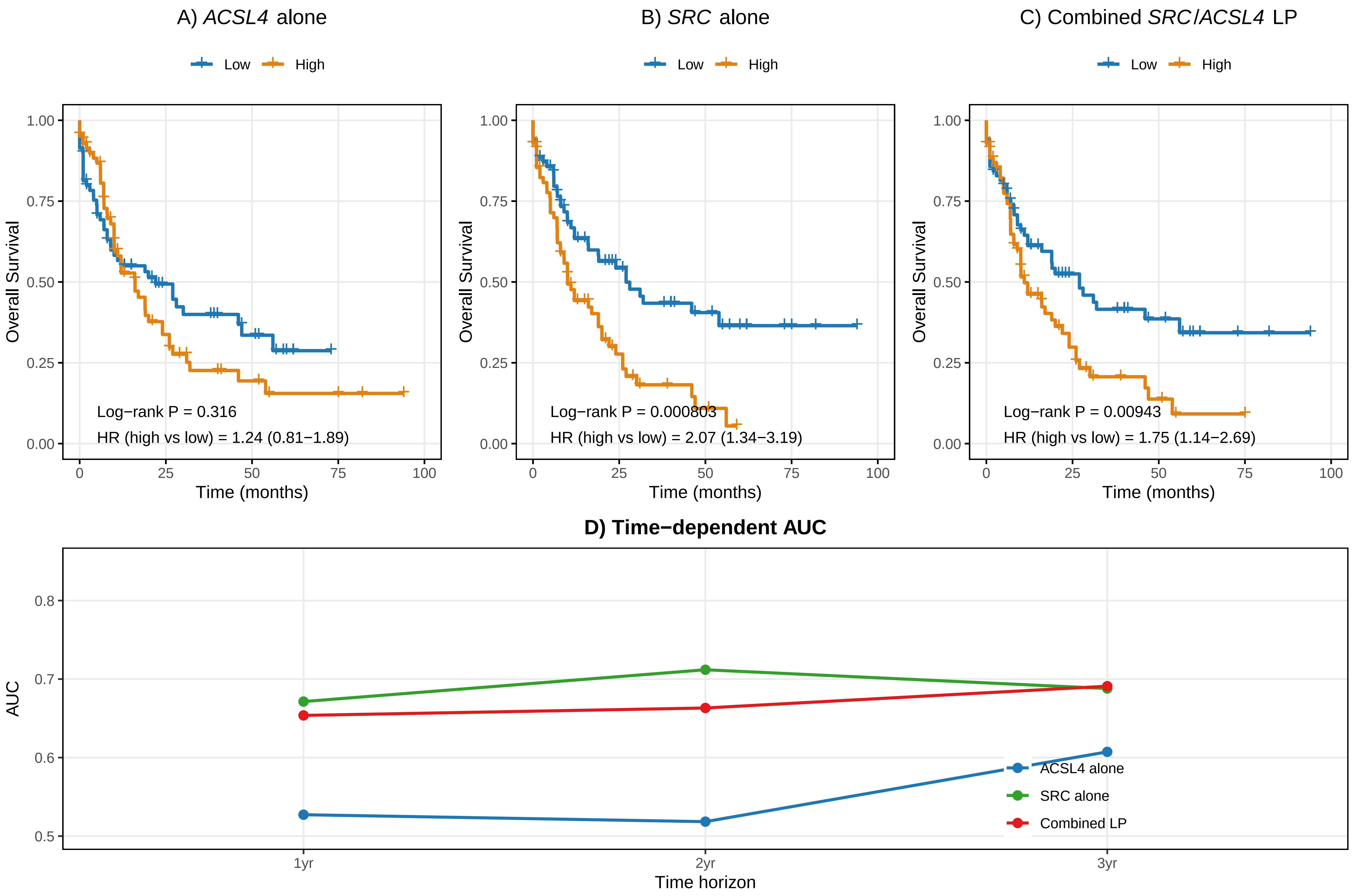

A) ACSL4 alone
Low
High
Overall Survival
1.00
0.75
0.50
0.25
0.00
0
25
50
75
100
Time (months)
Log−rank P = 0.316
HR (high vs low) = 1.24 (0.81−1.89)
B) SRC alone
Low
High
Log−rank P = 0.000803
HR (high vs low) = 2.07 (1.34−3.19)
C) Combined SRC/ACSL4 LP
Low
High
Log−rank P = 0.00943
HR (high vs low) = 1.75 (1.14−2.69)
D) Time−dependent AUC
AUC
0.8
0.7
0.6
0.5
1yr
2yr
3yr
Time horizon
ACSL4 alone
SRC alone
Combined LP

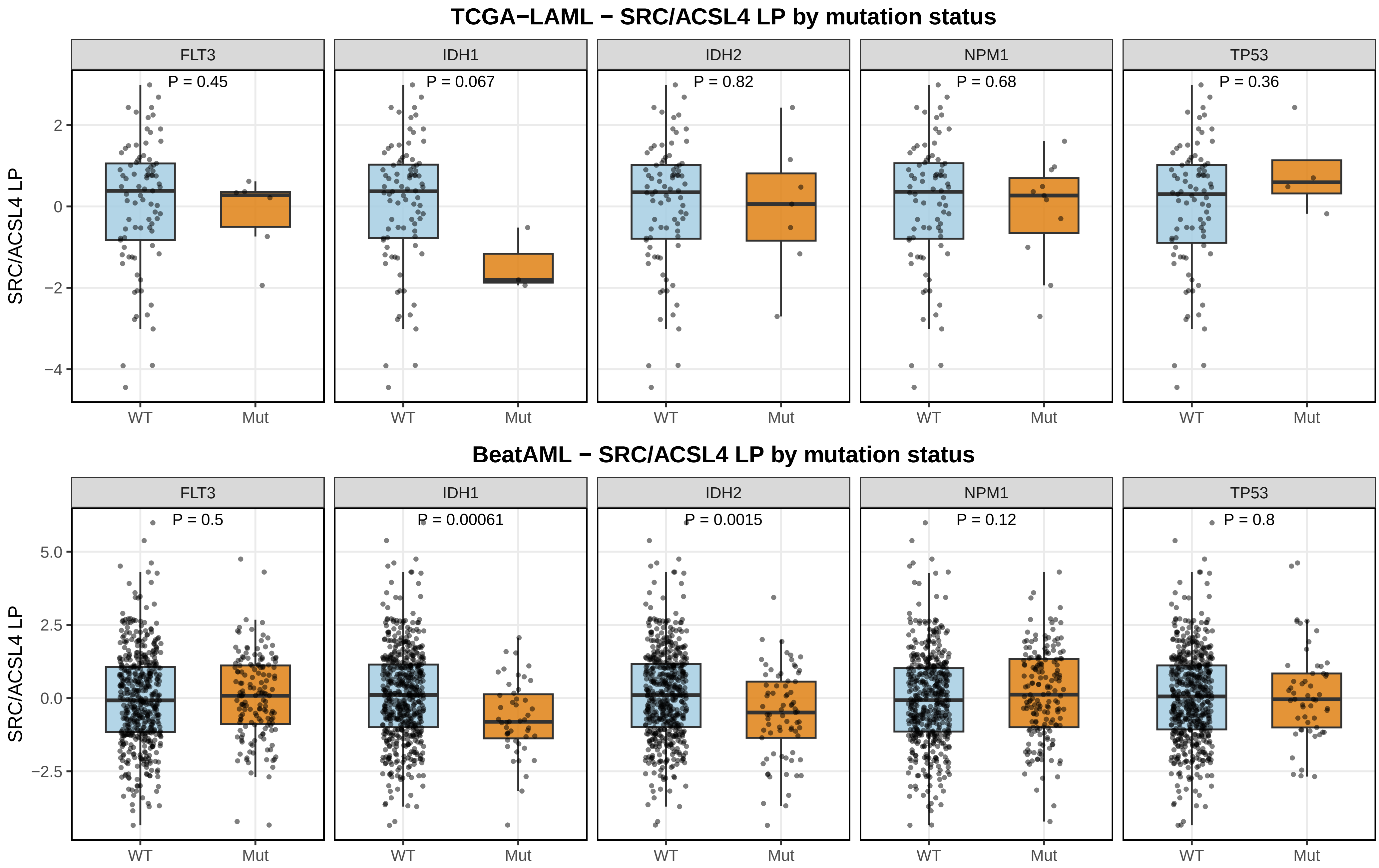

TCGA−LAML − SRC/ACSL4 LP by mutation status
FLT3
IDH1
IDH2
NPM1
TP53
P = 0.45
P = 0.067
P = 0.82
P = 0.68
P = 0.36
SRC/ACSL4 LP
WT
Mut
BeatAML − SRC/ACSL4 LP by mutation status
P = 0.5
P = 0.00061
P = 0.0015
P = 0.12
P = 0.8

Supplementary Table S1. LAMARCA−identified drug−gene co−dependencies in adult AML cell lines

Drug	MOA	OnTarget	CoTarget	N	Delta R^2	Partial rho	Partial rho P	FDR (co−target)	Z	Empirical P	LOLO median	LOLO frac >= 0	Random spec. P
A12B4C3	POLYNUCLEOTIDE KINASE/PHOSPHATASE INHIBITOR	PNKP	SQLE	15	0.633	0.741	0.00159	2.12e−04	5.35	0.00498	0.634	1.00	0.00498
TCN238	GLUTAMATE RECEPTOR POSITIVE ALLOSTERIC MODULATOR	GRM4	MMP8	15	0.597	−0.933	3.98e−07	2.09e−04	6.20	0.00498	0.599	1.00	0.00498
LDN−27219	TISSUE TRANSGLUTAMINASE INHIBITOR	TGM2	SLC25A5	15	0.592	−0.789	4.77e−04	2.65e−04	6.46	0.00498	0.598	1.00	0.00498
PANTOPRAZOLE	ATPASE INHIBITOR	ATP4A	ALKBH2	15	0.588	0.848	6.42e−05	2.65e−04	6.43	0.00498	0.593	1.00	0.00498
LASOFOXIFENE	SELECTIVE ESTROGEN RECEPTOR MODULATOR (SERM)	ESR1	HDAC1	15	0.584	−0.758	0.00106	2.24e−04	6.26	0.00995	0.577	1.00	0.00498
CINCHONINE	P GLYCOPROTEIN INHIBITOR	CYP2D6	TNKS	15	0.570	0.751	0.00125	3.16e−04	4.58	0.00995	0.571	1.00	0.00498
SU9516	CDK INHIBITOR	CDK1	EIF4E	15	0.568	0.812	2.34e−04	2.09e−04	5.85	0.00498	0.559	1.00	0.00498
LY2857785	CDK INHIBITOR	CDK9	GAMT	15	0.551	0.781	5.82e−04	5.05e−04	4.95	0.00995	0.550	1.00	0.00498
FRAX486	SERINE/THREONINE KINASE INHIBITOR	PAK1	F7	15	0.548	−0.758	0.00106	2.65e−04	4.18	0.00498	0.549	1.00	0.00498
ALPHA−ASARONE	CYTOCHROME P450 INHIBITOR, HMGCR INHIBITOR	HMGCR	SLC5A6	15	0.548	−0.652	0.00843	2.65e−04	3.85	0.0199	0.552	1.00	0.00498
CASIN	GTPASE INHIBITOR	CDC42	BRD1	15	0.545	−0.749	0.0013	2.65e−04	5.16	0.00498	0.547	1.00	0.005
K145	SPHINGOSINE KINASE INHIBITOR	SPHK2	CACNA2D4	15	0.543	−0.808	2.67e−04	2.21e−04	5.71	0.00498	0.529	1.00	0.005
SR−3677	RHO ASSOCIATED KINASE INHIBITOR	ROCK2	PTPN11	15	0.535	0.828	1.37e−04	2.12e−04	4.38	0.0149	0.537	1.00	0.00498
SUCROSE		ACTA1	KCNA10	15	0.527	−0.873	2.12e−05	8.16e−04	4.60	0.00498	0.525	1.00	0.00498
TC−S−7003	SRC INHIBITOR	LCK	GNRH1	15	0.494	0.822	1.70e−04	5.66e−04	4.75	0.00498	0.494	1.00	0.00498
NHI−2	LACTATE DEHYDROGENASE INHIBITOR	LDHA	OTC	15	0.489	0.715	0.00276	2.21e−04	3.78	0.00498	0.488	1.00	0.00498
4−DAMP	CHOLINERGIC RECEPTOR ANTAGONIST	CHRM1	SOD2	15	0.485	0.673	0.006	0.00129	3.84	0.0149	0.490	1.00	0.00995
AZM−475271	SRC INHIBITOR	SRC	ACSL4	15	0.479	−0.819	1.89e−04	7.53e−04	3.66	0.0199	0.489	1.00	0.00498
W−54011	ANAPHYLATOXIN CHEMOTACTIC RECEPTOR ANTAGONIST	C5AR1	BST1	15	0.479	−0.684	0.00489	0.00136	4.34	0.00498	0.477	1.00	0.00498
A−784168	TRANSIENT RECEPTOR POTENTIAL CHANNEL ANTAGONIST	TRPV1	SERPINC1	15	0.477	0.644	0.00951	0.00136	4.44	0.00498	0.478	1.00	0.00498
9−ANTHRACENECARBOXYLIC−ACID		ANO1	PTGDR	15	0.474	−0.557	0.0311	0.00136	3.84	0.0199	0.480	1.00	0.005
MERBARONE	TOPOISOMERASE INHIBITOR	TOP2A	CNR2	15	0.467	0.800	3.42e−04	2.69e−04	3.61	0.0199	0.477	1.00	0.00498
LY288513	CCK RECEPTOR ANTAGONIST	CCKBR	ITGB1	15	0.462	−0.792	4.31e−04	0.00136	5.19	0.00498	0.462	1.00	0.00498
GSK2110183	AKT INHIBITOR	AKT1	CA8	15	0.459	−0.762	9.59e−04	0.00118	4.72	0.00498	0.455	1.00	0.00498
A−987306	HISTAMINE RECEPTOR ANTAGONIST	CHRM2	SLC12A4	15	0.456	−0.564	0.0286	9.76e−04	3.36	0.0149	0.461	1.00	0.00498
AI−10−49	CORE BINDING FACTOR INHIBITOR	CBFB	PTGER1	15	0.455	0.745	0.00145	0.00144	4.08	0.00498	0.455	1.00	0.00995
FLUDROCORTISONE−ACETATE		AR	CACNA1H	15	0.453	−0.593	0.0198	7.85e−04	4.25	0.00995	0.449	1.00	0.00498
DDR1−IN−1	DISCOIDIN DOMAIN RECEPTOR INHIBITOR	DDR1	PGRMC1	15	0.441	0.863	3.43e−05	0.00136	3.69	0.0199	0.443	1.00	0.00498
A−804598	PURINERGIC RECEPTOR ANTAGONIST	P2RX7	PYGM	15	0.439	0.559	0.0303	0.00155	4.06	0.0199	0.444	1.00	0.00498
SEGESTERONE−ACETATE	PROGESTERONE RECEPTOR AGONIST	PGR	DCLK2	15	0.432	0.780	6.06e−04	2.65e−04	4.03	0.00995	0.422	1.00	0.005
INDEGLITAZAR	PPAR RECEPTOR AGONIST	NCOA1	ACSL4	15	0.428	−0.751	0.00126	0.00312	4.25	0.00995	0.439	1.00	0.00498
BW−B70C	LIPOXYGENASE INHIBITOR	ALOX5	GPR39	15	0.426	0.652	0.00846	0.00304	4.44	0.00498	0.420	1.00	0.00498
TYRPHOSTIN−AG−825	PROTEIN TYROSINE KINASE INHIBITOR	ERBB2	NOS3	15	0.426	0.777	6.62e−04	0.00188	4.03	0.00498	0.423	1.00	0.00498
LOSARTAN	ANGIOTENSIN RECEPTOR ANTAGONIST	AGTR1	NPY2R	15	0.422	0.762	9.60e−04	0.00136	3.86	0.0199	0.422	1.00	0.00498
MJ−15	CANNABINOID RECEPTOR ANTAGONIST	CNR1	ENPEP	15	0.419	−0.804	3.04e−04	0.00309	3.50	0.0149	0.414	1.00	0.00498
ER−50891	RETINOID RECEPTOR ANTAGONIST	RARA	GSK3A	15	0.413	−0.765	8.91e−04	0.00134	3.86	0.00498	0.422	1.00	0.00498
TCS−2002	GLYCOGEN SYNTHASE KINASE INHIBITOR	GSK3B	NAGK	15	0.412	−0.807	2.75e−04	2.12e−04	3.09	0.0199	0.414	1.00	0.00498
NS−1643	VOLTAGE−GATED POTASSIUM CHANNEL ACTIVATOR	KCNH2	FADS2	15	0.410	0.640	0.0102	0.00134	3.25	0.0199	0.413	1.00	0.00498
VU10010	ACETYLCHOLINE RECEPTOR ALLOSTERIC MODULATOR	CHRM4	NR1H4	15	0.404	0.669	0.00634	0.00114	3.81	0.00995	0.409	1.00	0.00498
PLX647	RECEPTOR TYROSINE PROTEIN KINASE INHIBITOR	CSF1R	PANX1	15	0.400	0.609	0.0159	8.16e−04	4.03	0.00995	0.405	1.00	0.00498
SUCCINOBUCOL	ANTIOXIDANT	VCAM1	PTGDR	15	0.393	0.664	0.00693	0.00309	3.52	0.0149	0.388	1.00	0.0149
ELACYTARABINE	ANTINEOPLASTIC AGENT	DCK	ADK	15	0.390	−0.569	0.0267	0.00113	3.61	0.0149	0.386	1.00	0.00498
AMMONIUM−PERFLUOROCAPRYLATE	PIM KINASE INHIBITOR	PIM1	SIRT1	15	0.382	−0.497	0.0597	0.00136	3.19	0.0348	0.375	1.00	0.00498
LUZINDOLE	MELATONIN RECEPTOR ANTAGONIST	MTNR1A	HCRT	15	0.379	−0.685	0.00484	8.16e−04	3.66	0.0149	0.385	1.00	0.00498
CLONIXIN	CYCLOOXYGENASE INHIBITOR	PTGS1	PIK3CD	15	0.338	0.687	0.00469	0.00602	3.46	0.0149	0.340	1.00	0.00498
GSK2334470	PHOSPHOINOSITIDE DEPENDENT KINASE INHIBITOR	AURKA	RPS6KA1	15	0.335	−0.646	0.00931	2.12e−04	3.26	0.0199	0.333	1.00	0.00498
CP−640186	ACETYL−COA CARBOXYLASE INHIBITOR	ACACA	FABP4	15	0.331	0.643	0.00978	0.00312	3.74	0.0149	0.337	1.00	0.00995
KRCA−0008	ALK INIHIBITOR	ALK	MC1R	15	0.328	−0.653	0.00828	0.00112	2.57	0.0299	0.333	1.00	0.005
SB−452533	TRPV ANTAGONIST	TRPV1	NAGS	15	0.305	0.570	0.0267	0.00978	2.94	0.0249	0.311	1.00	0.00995
CIS−ACONITIC−ACID		ACO2	BLVRB	15	0.264	−0.791	4.47e−04	0.00251	2.49	0.0249	0.274	1.00	0.00498

Top 50 high−confidence co−dependencies ranked by Delta R^2 (variance in drug AUC explained by the on−target/co−target gene pair beyond on−target alone). All entries pass FDR < 0.01 (co−target), permutation Empirical P < 0.05, leave−one−line−out median > 0, and random specificity P < 0.01. Drugs cited in the manuscript: AZM−475271 (SRC −> ACSL4) and INDEGLITAZAR (NCOA1 −> ACSL4).

 TCGA−LAML Cox proportional hazards models for overall survival

Model	HR	95% CI low	95% CI high	P	n
LP (univariable)	1.269	1.097	1.468	0.00138	140
LP + age	1.179	1.014	1.371	0.0321	140
LP + age + FLT3/NPM1/TP53	1.122	0.933	1.349	0.223	92
LP + age + cyto risk	1.182	1.000	1.398	0.0506	138
LP + age + FLT3/NPM1/TP53 + cyto	1.160	0.946	1.423	0.154	90
LP + age + FLT3/NPM1/TP53/IDH1/IDH2	1.066	0.872	1.303	0.533	91

LP = z(SRC) + z(ACSL4) per patient. The IDH−extended model (last row) confirms the LP estimate is not driven by IDH1/IDH2 status (HR 1.066, P = 0.533, n = 91 with complete annotation). Models with fewer covariates have higher n because cytogenetic risk and mutation calls are missing in some specimens.

Supplementary Table S2 − Page 2 of 3. Extended Cox coefficients (LP + age + FLT3 + NPM1 + TP53 + IDH1 + IDH2)

Term	HR	95% CI low	95% CI high	P	n
LP_z	1.066	0.872	1.303	0.533	91
age_years	1.057	1.031	1.083	1.14e−05	91
FLT3mut	3.706	1.395	9.844	0.00858	91
NPM1mut	2.020	0.898	4.546	0.0893	91
TP53mut	1.620	0.534	4.910	0.394	91
IDH1mut	0.595	0.133	2.652	0.495	91
IDH2mut	0.330	0.100	1.094	0.0698	91

Per−covariate hazard ratios from the IDH−extended Cox model (n = 91). FLT3−ITD is the dominant adverse covariate; IDH2 mutation shows a protective trend. After adjustment for both IDH genes the LP retains no significant prognostic effect, consistent with the LP capturing monocytic−lineage biology rather than IDH−driven primitive AML biology.

Supplementary Table S2 − Page 3 of 3. Wilcoxon rank−sum tests of LP / ACSL4 / SRC stratified by AML driver mutations

Cohort	Mutation	Marker	n mut	n WT	median (mut)	median (WT)	P	FDR
TCGA−LAML	FLT3	ACSL4 (z−score)	6	85	−0.272	0.124	0.477	0.715
TCGA−LAML	FLT3	SRC (z−score)	6	85	0.040	0.294	0.981	0.981
TCGA−LAML	FLT3	SRC/ACSL4 LP	6	85	0.274	0.383	0.447	0.715
TCGA−LAML	NPM1	ACSL4 (z−score)	11	80	−0.256	0.142	0.208	0.534
TCGA−LAML	NPM1	SRC (z−score)	11	80	−0.044	0.273	0.889	0.952
TCGA−LAML	NPM1	SRC/ACSL4 LP	11	80	0.268	0.359	0.683	0.932
TCGA−LAML	TP53	ACSL4 (z−score)	4	87	−0.246	0.124	0.839	0.952
TCGA−LAML	TP53	SRC (z−score)	4	87	0.515	0.145	0.212	0.534
TCGA−LAML	TP53	SRC/ACSL4 LP	4	87	0.593	0.301	0.358	0.671
TCGA−LAML	IDH1	ACSL4 (z−score)	3	88	−0.514	0.121	0.201	0.534
TCGA−LAML	IDH1	SRC (z−score)	3	88	−0.444	0.303	0.145	0.534
TCGA−LAML	IDH1	SRC/ACSL4 LP	3	88	−1.806	0.372	0.0667	0.534
TCGA−LAML	IDH2	ACSL4 (z−score)	7	84	−0.514	0.125	0.214	0.534
TCGA−LAML	IDH2	SRC (z−score)	7	84	0.779	0.185	0.329	0.671
TCGA−LAML	IDH2	SRC/ACSL4 LP	7	84	0.057	0.348	0.817	0.952
BeatAML	FLT3	ACSL4 (z−score)	155	492	−0.048	−0.044	0.288	0.36
BeatAML	FLT3	SRC (z−score)	155	492	0.298	0.088	0.0381	0.0715
BeatAML	FLT3	SRC/ACSL4 LP	155	492	0.082	−0.077	0.504	0.575
BeatAML	NPM1	ACSL4 (z−score)	174	471	0.185	−0.107	0.00504	0.0151
BeatAML	NPM1	SRC (z−score)	174	471	0.104	0.156	0.536	0.575
BeatAML	NPM1	SRC/ACSL4 LP	174	471	0.119	−0.070	0.116	0.158
BeatAML	TP53	ACSL4 (z−score)	53	542	−0.222	−0.013	0.0927	0.139
BeatAML	TP53	SRC (z−score)	53	542	0.312	0.129	0.0527	0.0878
BeatAML	TP53	SRC/ACSL4 LP	53	542	−0.039	0.057	0.796	0.796
BeatAML	IDH1	ACSL4 (z−score)	47	548	−0.482	0.049	0.00441	0.0151
BeatAML	IDH1	SRC (z−score)	47	548	−0.254	0.192	0.0069	0.0173
BeatAML	IDH1	SRC/ACSL4 LP	47	548	−0.807	0.111	6.11e−04	0.00917
BeatAML	IDH2	ACSL4 (z−score)	73	522	−0.336	0.057	0.00346	0.0151
BeatAML	IDH2	SRC (z−score)	73	522	−0.048	0.177	0.0111	0.0238
BeatAML	IDH2	SRC/ACSL4 LP	73	522	−0.492	0.103	0.0015	0.0112

All three markers (ACSL4 z−score, SRC z−score, SRC/ACSL4 LP) compared between mutated and wild−type cases for FLT3, NPM1, TP53, IDH1 and IDH2. P = Wilcoxon rank−sum P value; FDR = Benjamini−Hochberg adjustment within each cohort across all 5 mutations and 3 markers (15 tests). Significant findings (FDR < 0.05): in BeatAML, the LP is depleted in IDH1−mut (P = 6.11e−04, FDR = 0.009) and IDH2−mut (P = 1.50e−03, FDR = 0.011). TCGA−LAML mutation subgroups are too small for confident comparison (n_mut <= 11).

Supplementary Table S3. ACSL4 vs SRC independence in BeatAML (n = 476)

Analysis	Estimate	P value	n
Pearson r − ACSL4 vs dasatinib AUC (univariable)	−0.248	4.34e−08	476
Pearson r − SRC vs dasatinib AUC (univariable)	−0.147	0.0013	476
Partial Pearson r − ACSL4 controlling for SRC	−0.223	8.70e−07	476
Partial Pearson r − SRC controlling for ACSL4	−0.098	0.0322	476
Partial Spearman rho − ACSL4 controlling for SRC	−0.213	2.80e−06	476
Partial Spearman rho − SRC controlling for ACSL4	−0.115	0.0121	476
Joint LM coefficient − ACSL4_z (with SRC_z)	−13.54	8.70e−07	476
Joint LM coefficient − SRC_z (with ACSL4_z)	−5.84	0.0322	476
Joint LM R^2 (both genes)	0.070		476
Univariable R^2 − ACSL4 alone	0.061		476
Univariable R^2 − SRC alone	0.022		476

Joint linear model: dasatinib AUC ~ ACSL4_z + SRC_z (z−scored expression). Partial correlations control for the other gene. Higher AUC = greater drug resistance, so negative estimates indicate ACSL4−high or SRC−high blasts are more dasatinib−sensitive. ACSL4 retains a strong association with dasatinib AUC after adjustment for SRC, and the joint−model coefficient for ACSL4 is approximately 2.3−fold larger than that of SRC.
